# LC/MS-Based Metabolomics Reveals Chemical Variations of Two Broccoli Varieties in Relation to Their Anticholinesterase Activity: In vitro and In silico Studies

**DOI:** 10.1007/s11130-024-01161-2

**Published:** 2024-04-12

**Authors:** Rana M. Ibrahim, Riham A. El-Shiekh, Osama G. Mohamed, Ahmed A. Al-Karmalawy, Ashootosh Tripathi, Passent M. Abdel-Baki

**Affiliations:** 1 Pharmacognosy Department, Faculty of Pharmacy, Cairo University, Kasr-El-Ainy Street, Cairo, 11562 Egypt; 2 Natural Products Discovery Core, Life Sciences Institute, University of Michigan, Ann Arbor, MI 48109 USA; 3 Department of Pharmaceutical Chemistry, Faculty of Pharmacy, Horus University-Egypt, 34518 New Damietta, Egypt; 4 Pharmaceutical Chemistry Department, Faculty of Pharmacy, Ahram Canadian University, 6th of October City, Giza 12566 Egypt; 5 Department of Medicinal Chemistry, College of Pharmacy, University of Michigan, Ann Arbor, MI 48109 USA

**Keywords:** Alzheimer’s disease, Metabolomics, Food analysis, Molecular docking, UPLC-Q-TOF-MS

## Abstract

Broccoli is commonly consumed as food and as medicine. However, comprehensive metabolic profiling of two broccoli varieties, Romanesco broccoli (RB) and purple broccoli (PB), in relation to their anticholinergic activity has not been fully disclosed. A total of 110 compounds were tentatively identified using UPLC-Q-TOF-MS metabolomics. Distinctively different metabolomic profiles of the two varieties were revealed by principal component analysis (PCA). Furthermore, by volcano diagram analysis, it was found that PB had a significantly higher content of phenolic acids, flavonoids, and glucosinolates, indicating the different beneficial health potentials of PB that demonstrated higher antioxidant and anticholinergic activities. Moreover, Pearson’s correlation analysis revealed 18 metabolites, mainly phenolic and sulfur compounds, as the main bioactive. The binding affinity of these biomarkers to the active sites of acetyl- and butyryl-cholinesterase enzymes was further validated using molecular docking studies. Results emphasize the broccoli significance as a functional food and nutraceutical source and highlight its beneficial effects against Alzheimer’s disease.

**Supplementary Information:**

The online version contains supplementary material available at 10.1007/s11130-024-01161-2.

## Introduction

Alzheimer’s disease (AD) is an age-related neurodegenerative chronic illness that affects the elderly and progresses over time. AD is regarded as a major cause of dementia which is a prominent cause of disability globally and a significant public health burden with rising healthcare expenses. According to the most recent World Health Organization (WHO) data, there will be 139 million people living with dementia in 2050, up from the anticipated 55 million in 2019 [1]. The pathogenesis of AD is the subject of numerous theories, including cholinergic neuron dysfunction, amyloid (A*β*) protein deposits, *τ*-protein hyperphosphorylation, and metal dyshomeostasis [2]. According to the cholinergic hypothesis, increasing acetylcholine levels in the brain by blocking cholinesterase enzymes is an efficient therapeutic approach for treating AD symptoms. acetyl- (AChE) and butyryl-cholinesterase (BuChE) is inhibited from achieving this [3]. However, this therapy approach just addresses symptoms and does not significantly delay or stop the progression of the disease [4]. It has been established that oxidative stress-induced neuroinflammation contributes to the etiology of AD [5]. Therefore, it is advisable to give the patient sufficient antioxidants as an additional treatment to control and slow the progression of AD. Due to the intricacy of the disease, medications that alter the activity of a specific target may not be sufficient to stop AD’s progression. Therefore, it is more likely that the discovery of multi-targeted drugs that combine several pharmacological activities will lead to effective therapy.

The impact of nutrition on human health has received more attention in recent years. Among those plant products are the Brassica vegetables (Family Brassicaceae) which are widely acknowledged to have significant antioxidant activity and beneficial impact on human health [6]. *Brassica oleracea* L., commonly known as broccoli, is one of the most recognized species among this group of vegetables. Recently, increased consumption of broccoli by several folds was detected due to awareness about its nutritional content and health benefits. In addition, it is low in calories which makes it suitable to be incorporated in different dietary plans. The absence of hardness, peels, and seeds in broccoli makes it a suitable food that can be easily chewed by the elderly [7]. Broccoli is rich in polyphenols, flavonoids, glucosinolates, vitamins, minerals, and fibers [8]. *B. oleracea* was reported to exhibit several pharmacological activities, such as antibacterial [9], antifungal [10], antioxidant and antitumor [11]. *B. oleracea* L. is available in numerous distinct forms. Two of the most recognized varieties are *B. oleracea* var. *botrytis* [Romanesco broccoli (RB)] and *B. oleracea* L. var. *italica* Plenck [purple broccoli (PB)]. This study aimed to use the untargeted metabolomics strategy for a comprehensive understanding of the chemical profiles of RB and PB correlating them with their antioxidant [2,2′-azino-bis-3-ethylbenzthiazoline-6-sulfonic acid (ABTS), ferric reducing antioxidant power (FRAP) and 1,1-diphenyl-2-picrylhydrazyl (DPPH)] and anti-cholinesterase activities (AChE and BuChE inhibitory activities) for the first time. In addition, molecular docking simulations were used to examine the probable binding modes and intermolecular interactions of the identified compounds with AChE and BuChE active sites. These results may represent the first evidence of the multi-targeted potential of the two broccoli varieties and their detected metabolites against AD.

## Experimental

See supplementary file.

## Results and Discussion

### Biological Activities of Romanesco and Purple Broccolis

The antioxidant and anti-Alzheimer activities of both samples are summarized in Table S1. Where, PB sample showed better antioxidant results than RB as revealed by its lower IC_50_ values that were close to standard Trolox; 74 ± 2.46, 210.6 ± 6.56, and 63.09 ± 3.13 *vs.* 69.17 ± 3.15, 207.2 ± 8.19, and 20.5 ± 1.2 µg/ml as ABTS, FRAP, and DPPH assays, respectively. Acetyl and butyrylcholinesterase revealed that both broccoli samples had more selectivity index to AChE than BuChE, and PB showed the highest activity (Table S1).

### UPLC-Q-TOF-MS Metabolite Profiling

The methanolic extracts of RB and PB varieties were analyzed using UPLC-Q-TOF-MS in negative ion mode to obtain comprehensive and comparative metabolites fingerprinting and to investigate variations in both primary and secondary metabolites required to clarify their anticholinergic activity. According to the literature, broccoli is enriched in glucosinolates and phenolic compounds, which have better responses in the negative ionization mode, compared to the positive mode [12]. The representative base peak chromatograms of the two varieties are demonstrated in Supplementary Materials Fig. S1. In total, 110 compounds were identified in the methanolic extracts. The details on their accurate masses [M-H]^−^, molecular formulas, retention time, tentative identification, and chemical class are shown in Table S2. These compounds can be classified into 9 chemical classes, including, amino acids, carbohydrates, phenolic acids, glucosinolates, fatty acids, flavonoids, sesquiterpenes, lipids, and organic acids.

#### Phenolic Acids

Phenolic acids, including hydroxybenzoic and hydroxycinnamic acids, are commonly found in Brassicaceae vegetables, especially broccoli [13]. They exhibited a wide array of bioactivities, including antioxidant, neuroprotective, anti-inflammatory, and other health-promoting activities [14]. In broccoli, phenolic acids could be found free or conjugated as esters with sugars, flavonoids, organic acids, or other phenolic acids [13]. In agreement with the literature, a total of 25 phenolic acids were tentatively identified in the methanol extract of RB and PB samples and listed in Table S2 [13, 15, 16].

#### Flavonoids

Flavonoids particularly flavonols, are a major group of bioactive constituents in broccoli and are found mainly glycosylated and/or acylated with phenolic acids [17]. MS fragmentation identified the main aglycones as kaempferol, quercetin, or isorhamnetin [18]. The degree of glycosylation ranges from monoglucosides to pentaglucosides and their positions were mainly at C-3 and/or C-7 [19]. Tentatively and considering the reported literature, the hexoses involved are likely to be glucose, while the inter-glycosidic bond of the diglucoside is mainly sophoroside (1→2) [20]. Consequently, 11 flavonoids were annotated in the methanol extract of the two broccoli samples as non-acylated flavonoid glycosides. Among them, 3 kaempferol glycosides (28, 51, and 79), 5 quercetin glycosides (32, 36, 38, 50, and 70), and 2 isorhamnetin glycosides (42, 49, and 80) were detected (Table S2). Additionally, 11 flavonoids were acylated with hydroxycinnamic acid and were found to lose their acyl group in MS^2^, characterized by the loss of 176 Da for feruloyl-, 162 Da for caffeoyl-, 206 Da for sinapoyl-, and 146 Da for coumaroyl-derivatives, respectively [18]. Moreover, their MS^2^ fragmentation behaviors are characteristic of flavonol-3-O-(acyl)glycoside-7-*O*-glycosides and/or flavonol-3-*O*-(acyl)diglucosides, which are in agreement with previous studies on Brassica vegetables [20]. Flavonoids were reported to have neuroprotective benefits by boosting neuronal survival, tissue perfusion, and cerebral blood flow, and reducing ischemic-related apoptosis [21]. Furthermore, flavonoids have an anti-amyloidogenic activity and diminish the loss of dopaminergic neurons in the brain [21]. They could prevent reactive oxygen species formation and caspase-3 activity, reduce membrane and DNA damage, increase antioxidant enzyme activity, maintain calcium homeostasis and mitochondrial potential, suppress neuronal apoptosis, and induce autophagy [22].

#### Glucosinolates

Glucosinolates are a diverse group of sulfur-rich natural products extensively found in Brassicaceae vegetables and responsible for their pungent flavor. Ten glucosinolates (Table S2) were detected in the RB and PB samples. As previously reported, the MS^2^ spectra of glucosinolates displayed characteristic fragment ions resulting from the breakage occurring around the thioether bond, with cleavage of the alkyl, glucose, and sulfate chains including neutral loss of R-N = C = S from the parent ion and formation of a thioglucose fragment [23]. Glucosinolates have been reported to have a wide array of bioactivities, including antibacterial, antifungal, anticancer, antidiabetic, antioxidant, anti-inflammatory, cardioprotective, and neuroprotective [24].

#### Amino, Organic, Fatty acids, and Lipids

Lastly, 12 amino acids, 4 organic acids, as well as several fatty acids and lipids were annotated according to their characteristic fragmentation pathway and listed in Table S2 [25]. Amino acids, as nutrients, have been demonstrated to influence brain growth and maturation with subsequent effects in infants through their immunomodulatory and anti-inflammatory effects. They also could ameliorate injury-induced cognitive impairment and prevent the progression of Alzheimer’s [26]. Interestingly, dicarboxylic amino acids could be considered suitable candidates for the synthesis of potent acetyl- and butyryl-cholinesterase inhibitors due to the presence of three pharmacophoric groups (two carboxylic acids and one amine) [26]. Additionally, polyunsaturated fatty acids can ameliorate the cognitive deficits of Alzheimer’s by limiting amyloid polymerization in neuronal cells [27]. They could improve neuronal development, enhance cognitive functions, reduce apoptosis-like neuronal death by increasing the neuronal resistance against oxidative stress, and maintain the structural integrity of neural membranes, they also increase cortical acetylcholine levels and concurrently enhance both learning and memory [27].

To the best of our knowledge, this is the first comparative and comprehensive metabolite profiling of RB and PB coupled with chemometrics and computational analysis, which is intended to provide chemical-based evidence for their differential biological effect on Alzheimer’s key enzymes. Our analysis revealed the presence of various chemical classes in the analyzed extracts, confirming the enrichment of RB and PB in multifunctional nutraceuticals against neurodegenerative disease. However, to demonstrate the main metabolic differences and similarities between the two varieties, the distribution of detected metabolites in both sample types was visualized using the Venn diagram (Fig. S2). It was interesting that 30% of annotated metabolites were unique in the PB sample, whereas only 12.7% of the metabolites were specific to the RB sample. The intercept shows the number of compounds detected in both samples was 63 metabolites (57.3%), suggesting important differences in the chemical profiles of the two varieties (Fig. S2). It also demonstrated that PB extract has a greater number of unique compounds in comparison to RB extract, indicating PB extract contained the most varied number of chemical constituents which may have contributed to its higher antioxidant and anticholinergic effects. Thus, multivariate data analyses were performed based on the tentatively identified metabolites and their corresponding peak areas to provide deep insight into the chemical heterogeneity between the two broccoli varieties and to correlate the possible bioactive compounds to the anticholinesterase activity of the extracts.

### Metabolite Profiles Comparison and Differentiating Metabolites Analysis

Due to the chemical complexity, the differences between the two broccoli types were not clear from LC/MS chromatograms. Further analysis using PCA was performed to obtain more information about the intrinsic similarities and differences in their chemical profiles. The score plot (Fig. S3A) showed that the 3 biological replicates of the 2 samples were separated into two distant groups, where the PB sample was located at the far-left side of the plot, (negative PC1 values), while the RB sample was positioned on the far-right side (positive PC1 values). The Analysis of the PCA loading plot (Fig. S3B) showed the metabolites contributing to the variance in PCA scores with each metabolite denoted by its name. Accordingly, rutin (50), kaempferol 7-*O*-glucoside (79), caffeic acid (64), and glucobrassicin (25) were more enriched in the PB sample. On the other hand, the RB sample was characterized by a high abundance of sinapic acid (71), ferulic acid (52), stearic acid (102), glutamine (6), and PI (18:3/0:0) (88). This implies the possession of distinctive features and indicates a diverse chemical makeup in both broccoli varieties.

**Fig. 1:**
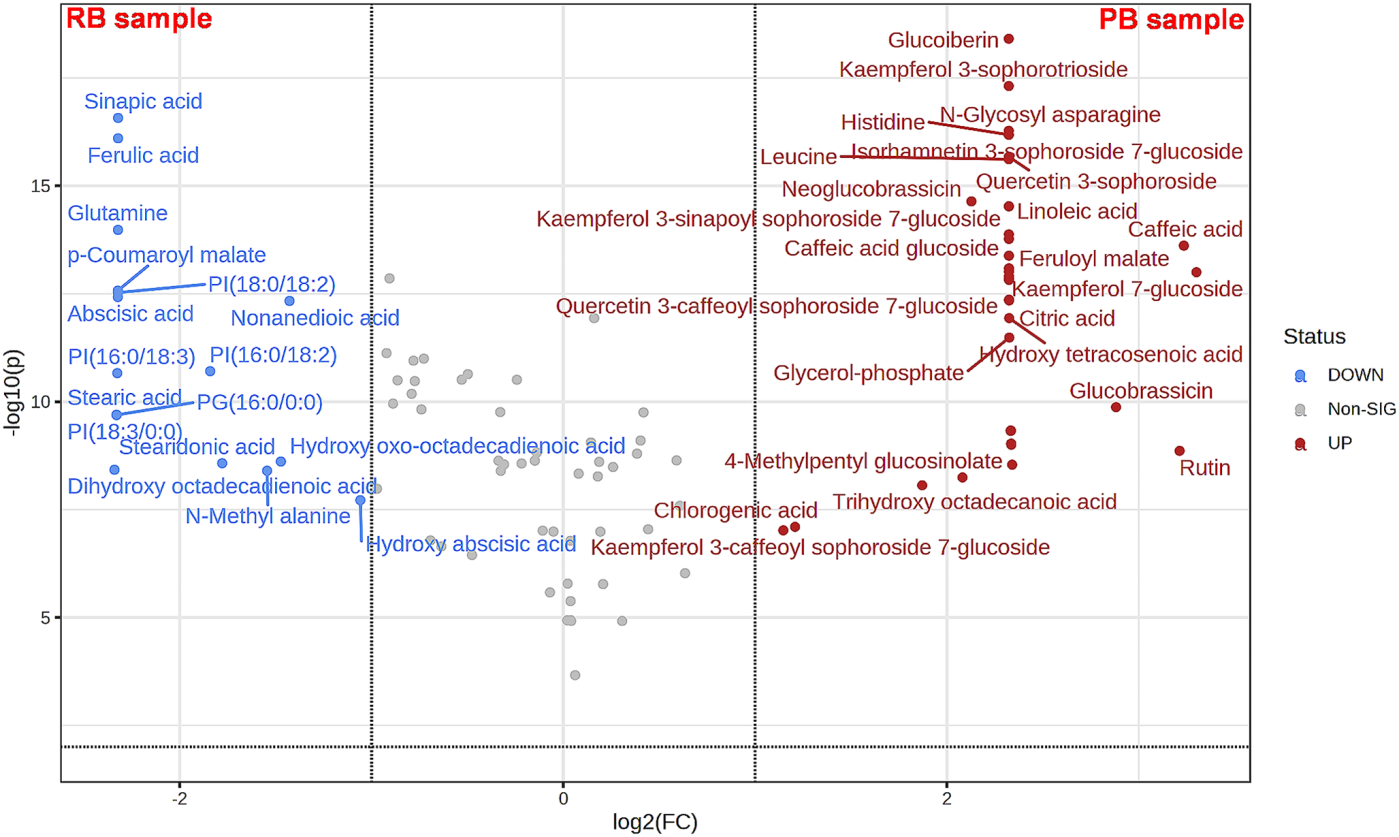
Volcano plot showed the significantly discriminating metabolites of two broccoli varieties (PB and RB). The negative logarithm of the *p*-value was used as the vertical axis (base 10), and the logarithm of fold change (base 2) between the two samples was used as the horizontal axis. Each point in the volcano plot represents a metabolite

Following that, a volcano plot was employed to visualize the differentiating metabolites between the two broccoli samples (Fig. 1). Metabolites having fold change ≥ 2.0 or ≤ 0.5 at p-value < 0.01 were regarded as the discriminating metabolites and thus colored as red (significantly higher in PB sample, fold change *≥* 2.0) or blue (significantly higher in RB sample, fold change *≤* 0.5). It can be seen that 60 metabolites were identified as the most relevant metabolites, of which 43 metabolites were significantly higher in the PB sample and 17 metabolites were significantly higher in the RB sample (Fig. 1 and Table S3). As can be observed in Fig. 1, PB demonstrated higher levels of phenolic and sulfur compounds, supporting its better antioxidant and anticholinergic effects.

### Metabolites-bioactivity Correlation

**Fig. 2:**
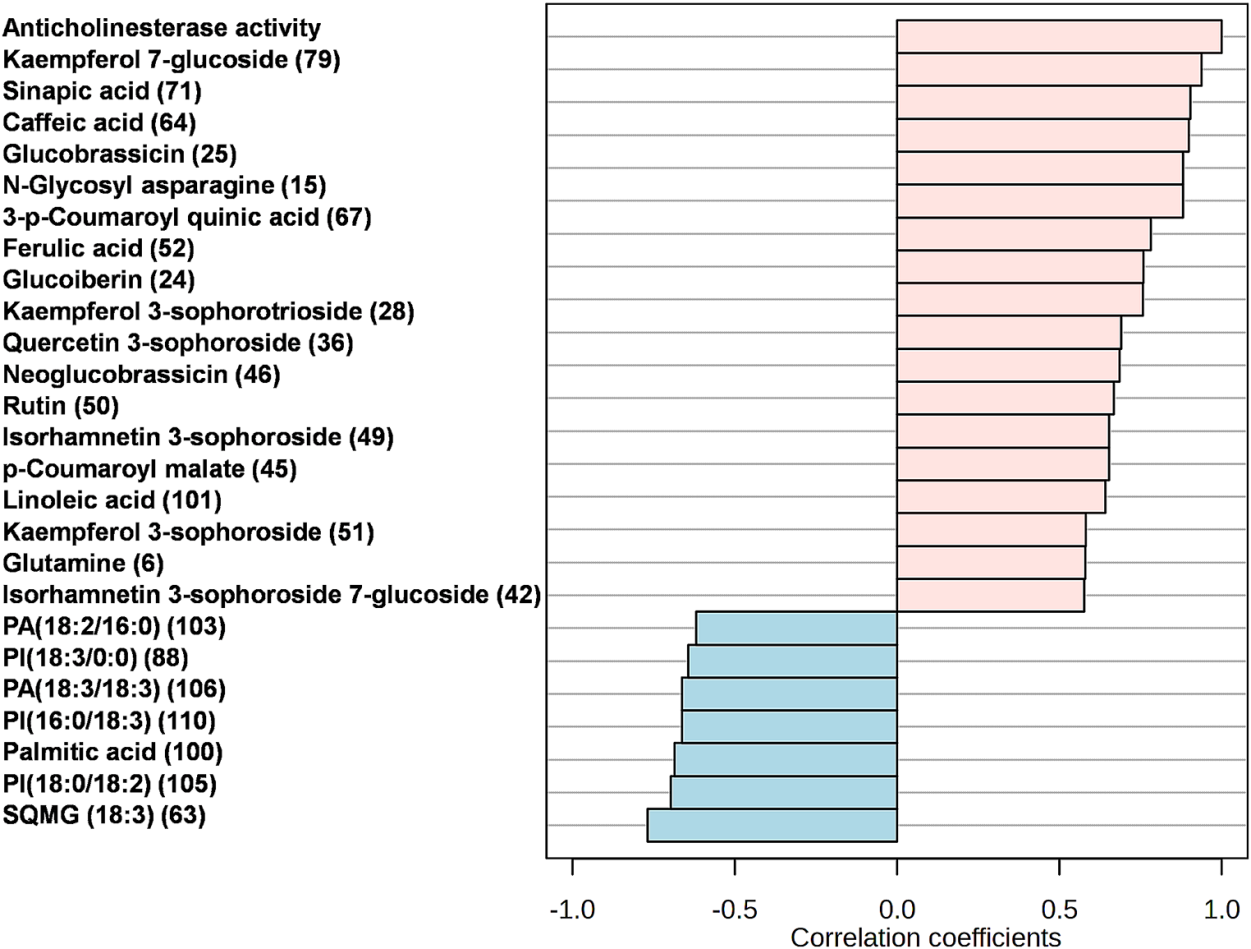
The top 25 metabolites correlated with the anticholinesterase activity of Romanesco and purple broccoli extracts (*n* = 3, *p* < 0.05). The details of the identified metabolites are recorded in Table S2

Pearson’s correlation analysis revealed certain relationships between broccoli metabolites and anticholinesterase activity, which were only considered for discussion when Pearson’s correlation coefficient (r) was > 0.5 at *p* < 0.01, and with false discovery rate (FDR) ≤ 0.01. The top 18 metabolites (Fig. 2) that were positively correlated with the activity were 7 flavonoids (79, 28, 36, 50, 49, 51, and 42), 5 phenolic acids (71, 64, 67, 52, and 45), 3 glucosinolates (25, 46, and 24); 2 amino acids (15 and 6), as well as linoleic acid (101). As can be observed, the majority of the correlated bioactive compounds belong to phenolic acid, flavonoid, and glucosinolate classes. The abundance of bioactive metabolites in the two varieties was visualized in box plots (Fig. S4), which clearly show that out of the 18 positively correlated metabolites, only four (71, 52, 45, and 6) predominated in the RP sample, indicating that PB had a wider range of bioactive metabolites, explaining its strong antioxidant and cholinesterase inhibitory effects. Interestingly, our results are in line with previous studies that reported the anti-Alzheimer and cognition-enhancing role of phenolic acids. For instance, the identified ferulic, caffeic, and sinapic acids were reported to protect the blood-brain barrier and brain structures [28]. They could ameliorate neurological deficits, inhibit inflammatory and apoptotic signaling pathways in the neurons, and decrease levels of inflammatory enzymes and mediators, as well as the key enzymes responsible for the progression of Alzheimer’s disease [28]. Flavonoids are known to interact with proteins implicated in protein kinase and lipid kinase signaling cascades related to cellular survival and death [21]. They can boost endogenous cellular antioxidant defenses *via* modulation of signaling cascades and gene expressions [21]. Rutin, as an example, was shown to modify the cognitive and various behavioral symptoms of neurodegenerative diseases [29]. Previous research on the anticholinergic effect of glucobrassicin and its derivatives suggested that glucosinolates might be useful medicinal agents with memory-stimulating properties [30]. Lastly, amino acids, such as glutamine, leucine, and alanine have neuroprotective effects and could improve cognitive function, and overall memory performance, hence decreasing Alzheimer’s progression [26]. As far as the authors know this is the first correlation of Romanesco and purple broccoli to their anti-Alzheimer’s potential. Further, a molecular docking study was performed for the detected biomarkers to investigate their potential binding modes and intermolecular interactions with AChE and BuChE that explain their inhibitory effects for the first time.

### Docking Studies

The 18 identified metabolites from both RB and PB using Pearson’s correlation analysis were docked against both AChE and BuChE target enzymes. This was done to validate their potential anti-Alzheimer therapeutic effects (Table 1) using drug design approaches [31, 32]. Furthermore, these metabolites were compared to the co-crystal inhibitor (donepezil) in each target receptor. The co-crystal of AChE (donepezil) was found to interact with Asp74, Phe295, and Trp286; however, the co-crystal of BuChE bound both Ser287 and Trp82. This is important to identify the key amino acids responsible for producing the antagonistic activity in each target enzyme. The docked co-crystallized inhibitors of AChE and BuChE achieved binding scores of -9.23 and − 8.89 kcal/mol, respectively. According to the binding scores (Table S4), it was clear that 3-*p*-coumaroyl quinic acid, glucobrassicin, and rutin were the frontier metabolites against both target receptors (AChE and BuChE). 3-*p*-Coumaroyl quinic acid achieved binding scores of -7.76 and − 6.78 kcal/mol against AChE and BuChE, respectively. It formed two hydrogen bonds with Asp74 and His447 of AChE, a hydrogen bond with Ser287, and a hydrogen-pi interaction with Trp82 of BuChE. On the other side, glucobrassicin got binding scores of -8.48 and − 7.95 kcal/mol against AChE and BuChE, respectively. Glucobrassicin formed two hydrogen bonds with Asp74 and Phe295, besides, a hydrogen-pi bond with Trp286 of AChE. Moreover, it formed three hydrogen bonds with Asp70, Gly116, and Thr120, besides, a hydrogen-pi bond with Trp82 of the BuChE receptor. Furthermore, the binding modes of rutin towards AChE and BuChE showed (one hydrogen bond with Asp74 and two hydrogen-pi interactions with Trp286) and (four hydrogen bonds with Ser287, Ser198, Glu197, and His438, and one pi-pi interaction with Trp82), respectively. Rutin binding scores were recorded to be -8.74 and − 8.97 kcal/mol towards AChE and BuChE, respectively. Accordingly, the three selected metabolites (3-*p*-coumaroyl quinic acid, glucobrassicin, and rutin) are highly recommended for more advanced studies as potential inhibitors for both AChE and BuChE. This is based on their promising binding scores which exceeded those of the co-crystallized inhibitors in some cases and similar binding modes which reflect the potential antagonistic activities as well.

**Table 1:** Docking scores of the selected metabolites

No	Compound	AChE	BuChE
1.	3-*p*-Coumaroyl quinic acid	-7.76283	-6.78487
2.	Caffeic acid	-5.65045	-5.04304
3.	Caffeic acid-3-glucoside	-7.61468	-6.90203
4.	Ferulic acid	-6.16812	-5.42254
5.	Glucobrassicin	-8.48737	-7.95914
6.	Glucoiberin	-8.12582	-7.72128
7.	Glutamine	-4.97057	-4.92143
8.	Isorhamnetin-3-*O*-sophoroside	-7.28908	-9.63232
9.	Kaempferol-3-*O*-sophoroside	-7.16402	-9.73025
10.	Kaempferol-3-*O*-sophorotrioside	-8.46265	-8.84134
11.	kaempferol-7-*O*-glucoside	-8.3638	-7.83776
12.	Linoleic acid	-7.89288	-7.01289
13.	Neoglucobrassicin	-8.21207	-8.54682
14.	*N*-Glycosyl-L-asparagine	-7.19152	-6.30433
15.	*p*-Coumaroyl malate	-6.68794	-5.92068
16.	Quercetin-3-*O*-sophoroside	-7.39075	-9.87217
17.	Rutin	-8.74668	-8.97952
18.	Sinapic acid	-6.41595	-6.0637
19.	Co-crystallized inhibitor	-9.23658	-8.89561

## Conclusions

In this study, an untargeted metabolomic approach based on UPLC-Q-TOF-MS was employed to reveal the chemical variation of RB and PB in relation to their antioxidant and anticholinergic effects. A total of 110 compounds were tentatively identified encompassing mainly, phenolic acids, flavonoids, glucosinolates, and lipids. Furthermore, as revealed by PCA and the volcano plot, distinctive differences in metabolic profiles between these two varieties of broccoli were found. PB revealed superior antioxidant and anticholinesterase activity over RB, primarily due to its enrichment in phenolic and sulfur compounds. Bioactive compounds positively correlated to the bioactivity were highlighted by Pearson’s correlation analysis and confirmed by molecular docking against both AChE and BuChE target enzymes. The results highlight the importance of broccoli as a functional food and medicine and reveal it as a promising candidate therapeutic agent for the prevention or treatment of Alzheimer’s disease. However, future research should focus on the isolation and structural elucidation of these bioactive markers, as well as the mechanism behind their synergistic interactions, and large-scale in vitro and in vivo investigations are necessary to confirm their efficacy and safety.

### Electronic Supplementary Material

Below is the link to the electronic supplementary material.

Supplementary Material 1

## Data Availability

No datasets were generated or analysed during the current study.

## Abbreviations

AChE: acetylcholinesterase
AD: Alzheimer’s disease
BuChE: butyryl-cholinesterase
PB: purple broccoli
PCA: principal component analysis
RB: Romanesco broccoli
