# Supplementary material for: LC/MS-Based Metabolomics Reveals Chemical Variations of Two Broccoli Varieties in Relation to Their Anticholinesterase Activity: In vitro and In silico Studies
Source: Plant Foods Hum Nutr. 2024 Apr 12;79(2):359–66. doi: 10.1007/s11130-024-01161-2 (PMC11178554; doi:10.1007/s11130-024-01161-2)
Supplement: Supplementary file 1 — Supplementary Material 1 [file 11130_2024_1161_MOESM1_ESM.docx]

**LC/MS-Based Metabolomics Reveals Chemical Variations of Two Broccoli Varieties in Relation to their Anticholinesterase Activity:** ***In vitro* and *In silico* Studies**

[Rana M. Ibrahim](https://pubmed.ncbi.nlm.nih.gov/?term=Ibrahim+RM&cauthor_id=36040657)^1^, Riham A. El-Shiekh^1^***, Osama G. Mohamed^1,2^, Ahmed A. Al-Karmalawy^3,4^, Ashootosh Tripathi^2,5^, [Passent M. Abdel-Baki](https://pubmed.ncbi.nlm.nih.gov/?term=Abdel-Baki+PM&cauthor_id=36040657)^1^***

^1^Pharmacognosy Department, Faculty of Pharmacy, Cairo University, Kasr-El-Ainy Street, Cairo, 11562, Egypt.

^2^Natural Products Discovery Core, Life Sciences Institute, University of Michigan, Ann Arbor, MI 48109, USA.

^3^Department of Pharmaceutical Chemistry, Faculty of Pharmacy, Horus University-Egypt, New Damietta 34518, Egypt.

^4^Pharmaceutical Chemistry Department, Faculty of Pharmacy, Ahram Canadian University, 6th of October City, Giza 12566, Egypt.

^5^Department of Medicinal Chemistry, College of Pharmacy, University of Michigan, Ann Arbor, MI 48109, USA.

** Authors to whom* *correspondence should be addressed.*

**Riham A. El-Shiekh,** Department of Pharmacognosy, Faculty of Pharmacy, Cairo University, Kasr el Aini st., Cairo, 11562, Egypt. E-mail address; [*riham.adel@pharma.cu.edu.eg*](mailto:riham.adel@pharma.cu.edu.eg)*.* [*https://orcid.org/*0000-0002-3179-3352](https://orcid.org/0000-0002-3179-3352).

[**Passent M. Abdel-Baki**](https://pubmed.ncbi.nlm.nih.gov/?term=Abdel-Baki+PM&cauthor_id=36040657)**,** Department of Pharmacognosy, Faculty of Pharmacy, Cairo University, Kasr el Aini st., Cairo, 11562, Egypt. E-mail address; [passent.mohamed@pharma.cu.edu.eg](mailto:passent.mohamed@pharma.cu.edu.eg). *https://orcid.org/*0000-0003-3809-3268

***Experimental design***

***Plant material and extraction***

The heads of *Brassica oleracea* var. *botrytis* (Romanesco broccoli (RB)) and *Brassica oleracea* var. *italica* (purple broccoli (PB)) were collected during the harvest season in February 2022 from Makar farm located on the Sakkara-road, Giza, Egypt. Prof. Abdel-Haleem Abdel-Motagaly, Department of Flora, Agriculture Museum authenticated the plants. Samples were kept in the Herbarium of the Pharmacognosy Department, Faculty of Pharmacy, Cairo University. Voucher specimen numbers were 10.04.2022II and 10.04.2022III. The heads were freeze-dried (-80 °C), lyophilized, powdered, and then extracted with methanol (2 L) till exhaustion. Solvents were removed with a rotary evaporator at 50 °C to obtain the extracts. For each variety, 3 samples (500 gm each) were extracted in parallel under the same conditions. Then 10 mg from each extract was transferred to an LC vial, dissolved in 1.5 mL methanol, and kept at -20 °C till further LC-MS analysis. For biological analysis, 20 mg of the dried methanolic extracts were dissolved in DMSO.

***Chemicals***

2,2’-azino-bis-3-ethylbenzthiazoline-6-sulphonic acid (ABTS), 1,1-diphenyl-2-picrylhydrazyl (DPPH), trolox, tripyridyl triazine (TPTZ), FeCl_3_, Tris-HCl buffer, dithio-bis-(2-nitrobenzoic acid (DTNB), Butyrylthiocholine iodide, acetylthiocholine, acetylcholinesterase (AChE) from Electric eel and butyrylcholinesterase (BuChE) from equine serum were purchased from Sigma Chemical Co. (St. Louis, MO, USA).

***Antioxidant activity***

Antioxidant activities of both samples were assessed using 2,2’-azino-bis-3-ethylbenzthiazoline-6-sulphonic acid (ABTS)[1], Ferric Reducing Antioxidant Power (FRAP) [2], and 1,1-diphenyl-2-picrylhydrazyl (DPPH) [3] antioxidant capacities.

Linear regression was performed for the calculation of the IC_50_ (50% inhibitory concentration), and Microsoft EXCEL 2010 program and graph pad instate 6.0 software were used for the data analysis.

% Inhibition = [1−(corrected A/corrected B)] * 100 (1).

***Cholinesterase inhibitory activity determination***

Cholinesterase inhibitory activities (AChE, and BuChE) were performed as described by the standard technique [4, 5]. The IC_50_ (50% inhibitory concentration) was calculated using linear regression and the data analysis was done using the Microsoft Excel 2010 and Graph Pad Instate 6.0 programs. % Inhibition = [(Reading B - Reading A / reading B) × 100].

**Statistical Analysis**

Data are presented as mean ± standard deviation (SD) after they were subjected to one way analysis of variance (ANOVA) followed by (Tukey) post hoc test at level of P ≤0.05.

UPLC-Q-TOF-MS

Ultra-high-performance liquid chromatograms (UPLC) were attained on an Agilent LC-MS system composed of an Agilent 1290 Infinity II UPLC coupled to an Agilent 6545 ESI-Q-TOF-MS in negative mode, aliquots (1 µL) of EtOAc extract (1 mg/mL in MeOH) were analyzed on a Kinetex phenyl-hexyl (1.7 μm, 2.1 × 50 mm) column eluted with 1 min isocratic elution of 90% A (A: 100% H_2_O + 0.1% formic acid) then 6 min linear gradient elution to 100% B (95% MeCN + 5% H_2_O + 0.1% formic acid) with a flow rate of 0.4 mL/min. ESI conditions were set with the capillary temperature at 320 °C, source voltage at 3.5 kV, and a sheath gas flow rate of 11 L/ min. Ions were detected in the full scan at an intensity above 1000 counts at 6 scans/s, with an isolation width of 1.3 ~m/z, a maximum of 9 selected precursors per cycle, and using ramped collision energy (5 × m/z/100 + 10 eV). Purine C_5_H_4_N_4_ [M+H]+ ion (m/z 121.0508) and hexakis (1H,1H,3H-tetrafluoropropoxy)-phosphazene C_18_H_18_F_24_N_3_O_6_P_3_ [M+H]+ ion (m/z 922.0098) were used as internal lock masses for positive mode while TFA C_2_HF_3_O_2_ [M−H]− ion (m/z 112.985587) and hexakis (1H,1H,3H-tetrafluoropropoxy)-phosphazene C_18_H_18_F_24_N_3_O_6_P_3_ [M+TFA−H]^−^ ion (*m/z* 1033.9881) were used as internal lock masses. Based on various online metabolites databases (Human Metabolome Database (http://www.hmdb.ca/), KEGG (<https://www.genome.jp/kegg/kegg1.html>), the Phytochemical Dictionary of Natural Product Database (https://dnp.chemnetbase.com/faces/chemical/ChemicalSearch.xhtml), LipidMaps (<https://www.lipidmaps.org/>), and MassBank of North America (https://mona.fiehnlab.ucdavis.edu/), compound identifications with the high level of confidence (MSI level 2) were achieved by matching experimental extract spectra against library retention times (RT), accurate precursor masses (m/z) and MS/MS spectra, taking into consideration similar acquisition parameters (e.g., resolution, collision energy, ionization, MS level) to ensure the validity of the match and decision criteria. More evidence for compound annotations was acquired through the diagnostic MS/MS fragments, ionization behavior, parent compound information, and reviewing the reported chemical constituents in broccoli [6-11].

Data Processing

Raw data of the three biological replicates acquired from the UPLC-QTOF-MS/MS was first converted to the mzXML format by msConvert software and then processed by using the mzmine 2.53 software for peak extraction and peak alignment [12]. The aligned feature list from the MZmine software was exported as a CSV matrix consisting of 6 columns (2 samples X 3 replicates) and 110 rows (identified features from the LC-MS dataset) and exported to the online platform MetaboAnalyst 5 (https://www.metaboanalyst.ca/MetaboAnalyst/ModuleView.xhtml) for further chemometric analysis, including principal component analysis (PCA) and volcano plot to identify the metabolites that significantly differentiated between the two broccoli varieties. For statistical significance, one-way ANOVA and Fisher’s least significant difference method (Fisher’s LSD) as a post-hoc analysis with *p* < 0.05 were selected. The most relevant metabolites correlated to the anticholinesterase activity were determined using Pearson’s correlations in the pattern search function.

***Docking studies***

The studied compounds were sketched in ChemDraw and transferred to the working window for correction and energy minimization steps [13]. Both AChE and BuChE target enzymes were downloaded through the Protein Data Bank website from <https://www.rcsb.org/structure/4EY7> and <https://www.rcsb.org/structure/8CGO>, respectively. Each target enzyme was corrected for errors, energy minimized, and 3D hydrogenated to be ready for the docking step [14, 15]. Then, the database of the prepared 18 metabolites was docked in the ligand site of each target enzyme using the co-crystal of each as a reference standard [16]. The best poses for the frontier compounds were selected based on the values of binding scores, RMSD (Root Mean Square Deviation), and binding modes as well [17].

Finally, the program validity was confirmed by redocking the co-crystallized inhibitor in each case within its binding site and obtaining low RMSD values (< 2 Å), besides, similar binding modes [18].

**Fig. S1.** The representative base peak chromatograms of the two varieties of broccoli were acquired in negative ionization mode. (A) Romanesco broccoli and (B) purple broccoli.


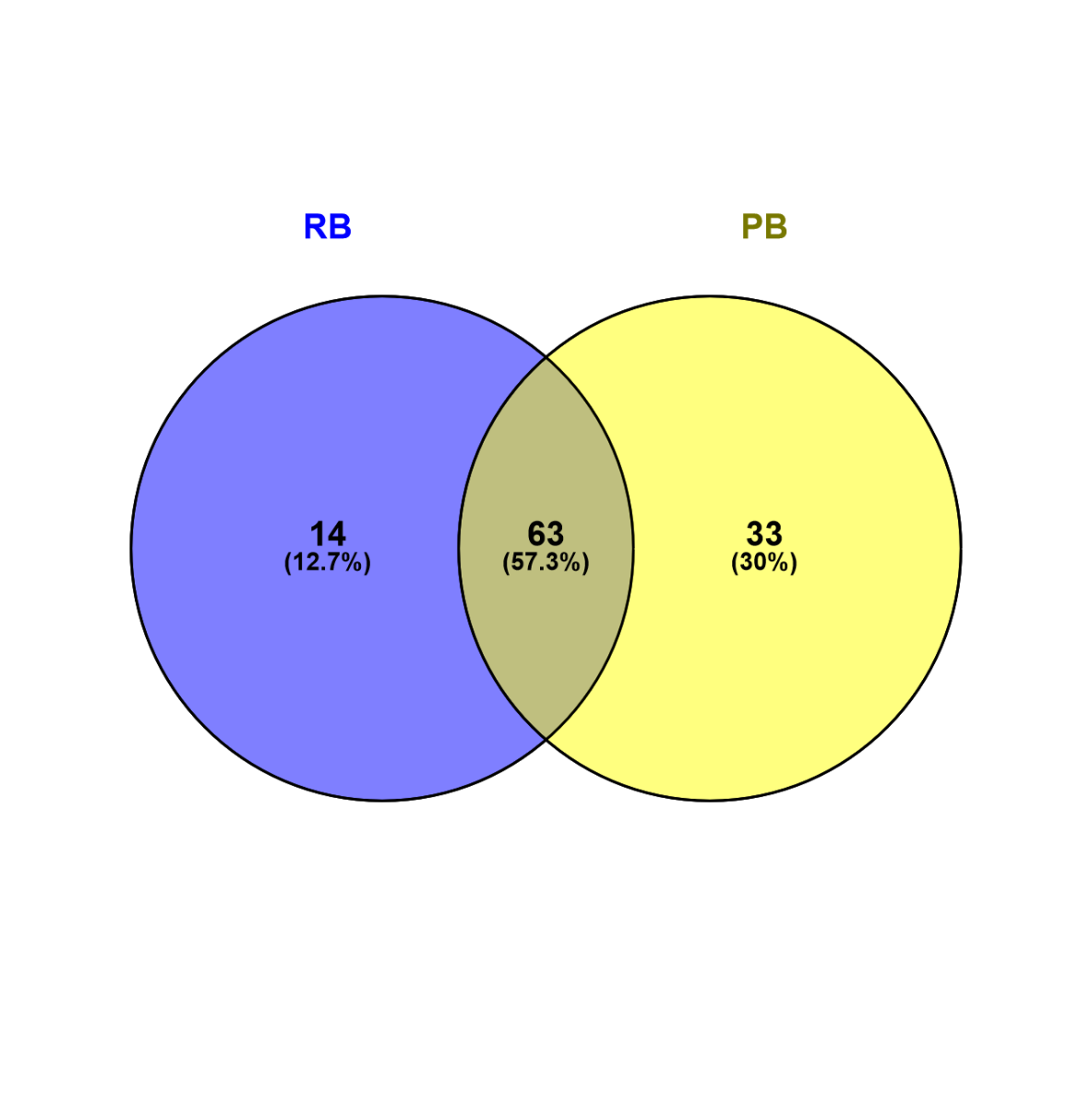


**Fig. S2.** Venn diagram showing the overlapping and unique metabolites in Romanesco (RB) and purple broccoli (PB).

**Fig. S3.** LC-MS-based PCA of the two broccoli varieties (*n* = 3). (**A**) Score and (**B**) loading plots for PC1 and PC2. The metabolites showing the largest absolute score values along each PC are named and their details are present in Table S2.

**Fig. S4.** Box plots present the relative abundance of the positively correlated metabolites with the anticholinesterase activity of broccoli extracts (r>0.5 at *p*<0.01). The y-axis represents the scaled and log_10_-transformed values of metabolite abundance.

**Table S1.** *In vitro* antioxidant and anti-Alzheimer activities of purple broccoli (PB) and Romanesco broccoli (RB) as compared to standard.

| Samples | **ABTS** | **FRAP** | **DPPH** | **AChE** | **BuChE** |
| --- | --- | --- | --- | --- | --- |
|  | **IC_50_ (μg/ml)** | | | | |
| Purple broccoli | 74±2.46^a^ | 210.6±6.56 ^a^ | 63.09±3.13 ^b^ | 6.55±0.75 ^b^ | 38.41±2.88 ^b^ |
| Romanesco broccoli | 80.91±3.71^b^ | 229.3±4.57 ^b^ | 88.54±2.05 ^c^ | 14.60±1.54 ^c^ | 46.26±3.90 ^c^ |
| Trolox | 69.17±3.15^a^ | 207.2±8.19 ^a^ | 20. 5 ± 1.2 ^a^ | - | - |
| Donepezil |  |  |  | 0.23 ± 0.03 ^a^ | 0.3 ± 0.0033 ^a^ |

There are no significant differences between the same letters in the same column at (*P* ≤ 0.05).

**Table S2.** Compounds identified in the methanol extracts of Romanesco (RB) and purple (PB) broccoli by UPLC-QTOF-MS/MS in negative ionization mode.

| **Peak no.** | **RT min** | **Molecular ion [M-H]^–^** | **Error ppm** | **MS/MS (*m*/*z*)** | **Elemental composition** | **Identification** | **Chemical class** | **Source** | |
| --- | --- | --- | --- | --- | --- | --- | --- | --- | --- |
|  |  |  |  |  |  |  |  | **RB** | **PB** |
| 1 | 0.36 | 173.1037 | -3.9 | 131, 87, 70, 58 | C_6_H_14_N_4_O_2_ | Arginine | Amino acid | + | + |
| 2 | 0.37 | 154.0607 | -9.5 | 110, 93, 81, 71 | C_6_H_9_N_3_O_2_ | Histidine | Amino acid | - | + |
| 3 | 0.37 | 214.0509 | 10.8 | 158, 144, 123, 93, 78 | C_5_H_14_NO_6_P | Glycerophospho-ethanolamine | Lipid | - | + |
| 4 | 0.37 | 341.1077 | -5.6 | 179, 167, 140, 137, 89 | C_12_H_22_O_11_ | Disaccharide | Carbohydrate | + | + |
| 5 | 0.38 | 133.0136 | -5.2 | 115, 71 | C_4_H_6_O_5_ | Malic acid | Organic acid | + | + |
| 6 | 0.39 | 145.0614 | -3 | 82 | C_5_H_10_N_2_O_3_ | Glutamine | Amino acid | + | - |
| 7 | 0.40 | 102.0553 | -7.1 | 84, 54 | C_4_H_9_NO_2_ | N-Methyl alanine | Amino acid | - | + |
| 8 | 0.40 | 104.0346 | -6.6 | 74 | C_3_H_7_NO_3_ | Serine | Amino acid | + | + |
| 9 | 0.41 | 116.0711 | -4.9 | 51 | C_5_H_11_NO_2_ | Valine | Amino acid | + | + |
| 10 | 0.41 | 118.0501 | -4.7 | 74 | C_4_H_9_NO_3_ | Threonine | Amino acid | + | + |
| 11 | 0.42 | 131.0454 | -6.0 | 114, 95, 72, 58 | C_4_H_8_N_2_O_3_ | Asparagine | Amino acid | + | + |
| 12 | 0.42 | 209.0783 | 4.6 | 194, 181, 165, 150 | C_10_H_10_O_5_ | Hydroxy ferulic acid | Phenolic acid | + | + |
| 13 | 0.43 | 203.0817 | -4.3 | 116, 75, 65 | C_11_H_12_N_2_O_2_ | Tryptophan | Amino acid | + | + |
| 14 | 0.43 | 195.0533 | -6.9 | 149, 129, 117, 87 | C_6_H_12_O_7_ | Gluconic acid | Carbohydrate | + | + |
| 15 | 0.44 | 293.0984 | -2.1 | 131, 113 | C_10_H_18_N_2_O_8_ | N-Glycosyl asparagine | Amino acid | - | + |
| 16 | 0.45 | 191.019 | -3.5 | 111, 87, 76, 57 | C_6_H_8_O_7_ | Citric acid | Organic acid | - | + |
| 17 | 0.47 | 191.0567 | 1.1 | 165, 155, 147, 127, 109 | C_7_H_12_O_7_ | Quinic acid | Organic acid | + | + |
| 18 | 0.51 | 130.0846 | 2.1 | 75, 71, 51 | C_6_H_13_NO_2_ | Leucine | Amino acid | - | + |
| 19 | 0.56 | 164.071 | -7.1 | 164, 147, 103, 92, 62 | C_9_H_11_NO_2_ | Phenylalanine | Amino acid | + | + |
| 20 | 0.57 | 205.0348 | -2.7 | 161, 87 | C_7_H_10_O_7_ | Methyl citric acid | Organic acid | - | + |
| 21 | 0.92 | 299.076 | -4.1 | 219, 137, 93 | C_13_H_16_O_8_ | Hydroxy benzoic acid glucoside | Phenolic acid | + | + |
| 22 | 0.93 | 137.0238 | -4.3 | 117, 109, 93 | C_7_H_6_O_3_ | Hydroxy benzoic acid | Phenolic acid | + | + |
| 23 | 1.03 | 358.0259 | -1.8 | 259, 200, 179, 164, 116, 96, 74 | C_10_H_17_NO_9_S_2_ | Sinigrin | Glucosinolate | + | + |
| 24 | 1.04 | 422.0272 | 4.1 | 341, 274, 259, 245, 225, 195, 180, 165, 96 | C_11_H_21_NO_10_S_3_ | Glucoiberin | Glucosinolate | + | + |
| 25 | 1.05 | 447.0516 | -4.7 | 259, 195, 97, 96, 80, 75 | C_16_H_20_N_2_O_9_S_2_ | Glucobrassicin | Glucosinolate | + | + |
| 26 | 1.09 | 153.0182 | -1.7 | 109 | C_7_H_6_O_4_ | Protocatechuic acid | Phenolic acid | + | + |
| 27 | 1.19 | 315.0742 | 2.2 | 175, 152, 109 | C_13_H_16_O_9_ | Protocatechuic acid glucoside | Phenolic acid | + | + |
| 28 | 1.23 | 771.1917 | -2.3 | 609, 446, 284, 179, 140 | C_33_H_40_O_21_ | Kaempferol 3-sophorotrioside | Flavonoid | - | + |
| 29 | 1.24 | 949.3132 | 5.4 | 865, 607, 462, 300, 161 | C_42_H_46_O_25_ | Quercetin 3-caffeoyl sophoroside 7-glucoside | Flavonoid | - | + |
| 30 | 1.27 | 917.2369 | 1.7 | 840, 721, 580, 476, 363, 284, 255, 178, 163 | C_42_H_46_O_23_ | Kaempferol 3-coumaroyl sophoroside 7-glucoside | Flavonoid | - | + |
| 31 | 1.32 | 963.2316 | -4.6 | 914, 801, 625, 518, 462, 300, 175, 163 | C_43_H_48_O_25_ | Quercetin 3-feruloyl sophoroside 7-glucoside | Flavonoid | + | + |
| 32 | 1.68 | 771.1999 | 1.5 | 485, 608, 574, 463, 299, 271, 254, 108 | C_33_H_40_O_21_ | Quercetin 3-rutinoside-7-glucoside | Flavonoid | - | + |
| 33 | 1.5 | 487.1479 | 3.5 | 325, 163, 145, 119 | C_21_H_28_O_13_ | p-Coumaric acid diglucoside | Phenolic acid | + | + |
| 34 | 1.5 | 339.0742 | 2.2 | 223, 208, 179, 164, 149, 133 | C_15_H_16_O_9_ | Sinapoyl malate | Phenolic acid | + | + |
| 35 | 1.59 | 372.0453 | 2.3 | 322, 259, 241, 178, 127, 96, 81, 74 | C_11_H_19_NO_9_S_2_ | Gluconapin | Glucosinolate | + | + |
| 36 | 1.45 | 625.1452 | 2.9 | 462, 299, 275, 205 | C_27_H_30_O_17_ | Quercetin 3-sophoroside | Flavonoid | - | + |
| 37 | 1.77 | 402.0539 | 1.8 | 259, 195, 161, 128, 96 | C_12_H_21_NO_10_S_2_ | 2-Hydroxy-4-pentenyl glucosinolate (Gluconapoleiferin | Glucosinolate | + | + |
| 38 | 1.91 | 787.1905 | -2.4 | 751, 711, 625, 462, 301, 300 | C_33_H_40_O_22_ | Quercetin 3-sophoroside 7-glucoside | Flavonoid | - | + |
| 39 | 1.94 | 933.2367 | 3.1 | 902, 609, 469, 447, 375, 284, 251, 215, 179, 172, 161 | C_42_H_46_O_24_ | Kaempferol 3-caffeoyl sophoroside 7-glucoside | Flavonoid | - | + |
| 40 | 2.09 | 831.2017 | 3.4 | 710, 601, 452, 315, 246, 175, 121 | C_38_H_40_O_21_ | Isorhamnetin 3-feruloyl sophoroside | Flavonoid | - | + |
| 41 | 2.15 | 374.0576 | -2.1 | 312, 276, 247, 214, 180, 150, 96, 74 | C_11_H_21_NO_9_S_2_ | Butyl glucosinolate | Glucosinolate | + | + |
| 42 | 2.2 | 801.2077 | -3.1 | 693, 493, 478, 449, 314, 251, 178 | C_34_H_42_O_22_ | Isorhamnetin 3-sophoroside 7-glucoside | Flavonoid | - | + |
| 43 | 2.3 | 503.1397 | -1.6 | 340, 257, 150 | C_21_H_28_O_14_ | Caffeic acid diglucoside | Phenolic acid | + | + |
| 44 | 2.36 | 547.1677 | 2.9 | 383, 223, 206, 190, 164 | C_23_H_32_O_15_ | Sinapic acid diglucoside | Phenolic acid | + | + |
| 45 | 2.42 | 279.0607 | 6.1 | 163, 133, 119, 71 | C_13_H_12_O_7_ | *p*-Coumaroyl malate | Phenolic acid | + | - |
| 46 | 2.45 | 477.062 | -4.8 | 298, 231, 171, 97, 96, 80 | C_17_H_22_N_2_O_10_S_2_ | Neoglucobrassicin | Glucosinolate | - | + |
| 47 | 2.54 | 325.1026 | 1.8 | 163, 119 | C_15_H_18_O_8_ | *p*-Coumaric acid glucoside | Phenolic acid | + | + |
| 48 | 2.61 | 341.0881 | 1.3 | 306, 241, 181, 96 | C_15_H_18_O_9_ | Caffeic acid glucoside | Phenolic acid | - | + |
| 49 | 2.61 | 639.151 | -8.8 | 476, 313, 285 | C_28_H_32_O_17_ | Isorhamnetin 3-sophoroside | Flavonoid | - | + |
| 50 | 2.69 | 609.2056 | 3.1 | 446, 299 | C_27_H_30_O_16_ | Rutin | Flavonoid | - | + |
| 51 | 2.71 | 609.1431 | -4.7 | 447, 284, 283 | C_27_H_30_O_16_ | Kaempferol 3-sophoroside | Flavonoid | - | + |
| 52 | 2.73 | 193.0517 | 1.5 | 178, 160, 134 | C_10_H_10_O_4_ | Ferulic acid | Phenolic acid | + | - |
| 53 | 2.75 | 947.2471 | 2.7 | 785, 446, 284, 257, 217, 193, 175, 161, 151 | C_43_H_48_O_24_ | Kaempferol 3-feruloyl sophorotrioside | Flavonoid | + | + |
| 54 | 2.8 | 977.2512 | -5.7 | 578, 446, 352, 284, 223, 205, 169, 134 | C_44_H_50_O_25_ | Kaempferol 3-sinapoyl sophoroside 7-glucoside | Flavonoid | - | + |
| 55 | 2.82 | 755.1861 | 2.3 | 699, 480, 285, 146, 109 | C_36_H_36_O_18_ | Kaempferol 3-coumaroyl sophoroside | Flavonoid | + | + |
| 56 | 2.85 | 187.0992 | 8.7 | 167, 125, 116, 70 | C_9_H_16_O_4_ | Nonanedioic acid | Fatty acid | + | + |
| 57 | 2.85 | 355.1040 | 1.8 | 258, 193, 178, 134 | C_16_H_20_O_9_ | Ferulic acid glucoside | Phenolic acid | - | + |
| 58 | 2.88 | 385.1139 | 1.2 | 223, 179, 164, 149, 119 | C_17_H_22_O_10_ | Sinapic acid glucoside | Phenolic acid | + | + |
| 59 | 2.91 | 371.0991 | 2.0 | 163, 119 | C_16_H_20_O_10_ | Hydroxy ferulic acid glucoside | Phenolic acid | + | + |
| 60 | 3.03 | 163.0389 | -1.6 | 119 | C_9_H_8_O_3_ | *p*-Coumaric acid | Phenolic acid | + | + |
| 61 | 3.05 | 309.0666 | 2.9 | 193, 133 | C_14_H_14_O_8_ | Feruloyl malate | Phenolic acid | - | + |
| 62 | 3.07 | 376.1134 | 4.1 | 193, 173, 155, 134 | C_17_H_20_O_9_ | Feruloyl quinic acid | Phenolic acid | + | + |
| 64 | 3.16 | 179.0355 | 2.6 | 134 | C_9_H_8_O_4_ | Caffeic acid | Phenolic acid | + | + |
| 65 | 3.23 | 263.1280 | -3.3 | 219, 204 | C_15_H_20_O_4_ | Abscisic acid | Sesquiterpene | + | + |
| 66 | 3.54 | 319.0755 | -7.9 | 275, 257, 173, 161, 119 | C_16_H_16_O_7_ | p-Coumaroyl shikimate | Phenolic acid | + | - |
| 67 | 3.48 | 337.0918 | -3.4 | 191, 163, 119 | C_16_H_18_O_8_ | 3-p-Coumaroyl quinic acid | Phenolic acid | - | + |
| 68 | 3.28 | 201.1126 | -3.0 | 183, 156, 140, 139, 79, 75 | C_10_H_18_O_4_ | Sebacic acid | Fatty acid | + | + |
| 69 | 3.55 | 785.20010 | 1.1 | 714, 462, 284, 221, 193, 161, 116, 103 | C_37_H_38_O_19_ | Kaempferol 3-feruloyl sophoroside | Flavonoid | - | + |
| 70 | 3.60 | 463.0866 | -3.4 | 301, 255 | C_21_H_20_O_12_ | Quercetin-7-glucoside | Flavonoid | + | + |
| 71 | 3.72 | 223.0703 | 1.8 | 149 | C_11_H_12_O_5_ | Sinapic acid | Phenolic acid | + | - |
| 72 | 3.73 | 845.2128 | -2.3 | 665, 477, 314, 223, 209, 198, 101 | C_39_H_42_O_21_ | Isorhamnetin 3-sinapoyl sophoroside | Flavonoid | - | + |
| 73 | 3.74 | 295.0894 | 2.4 | 179, 175, 160, 147, 133 | C_13_H_12_O_8_ | Caffeoyl malate | Phenolic acid | - | + |
| 74 | 3.82 | 386.0591 | 1.5 | 343, 197, 96 | C_12_H_21_NO_9_S_2_ | 1-Pentenyl glucosinolate | Glucosinolate | + | + |
| 75 | 3.84 | 402.0919 | 5.3 | 392, 248, 259, 173, 195, 121, 97, 80 | C_13_H_25_NO_9_S_2_ | 4-Methylpentyl glucosinolate | Glucosinolate | + | + |
| 76 | 3.85 | 815.2097 | -3.1 | 680, 591, 284, 205, 190, 180, 150, 114 | C_38_H_40_O_20_ | Kaempferol 3-sinapoyl sophoroside | Flavonoid | + | + |
| 77 | 3.86 | 353.0885 | 1.6 | 191, 179, 161, 135 | C_16_H_18_O_9_ | Chlorogenic acid | Phenolic acid | - | + |
| 78 | 3.89 | 463.0494 | 1.8 | 426, 393, 323, 291, 259, 206, 169, 116, 96 | C_16_H_20_N_2_O_10_S_2_ | 4-Hydroxyglucobrassicin | Glucosinolate | + | + |
| 79 | 3.89 | 447.0931 | -2.6 | 285, 257, 229, 177, 151 | C_21_H_20_O_11_ | Kaempferol 7-glucoside | Flavonoid | + | + |
| 80 | 3.91 | 461.1132 | 5.6 | 313, 299, 261, 245 | C_22_H_22_O_11_ | Isorhamnetin 7-rhamnoside | Flavonoid | + | + |
| 81 | 3.93 | 211.1331 | -4.0 | 142, 97, 69 | C_12_H_20_O_3_ | Traumatin | Fatty acid | + | + |
| 82 | 3.99 | 229.1435 | -4.4 | 211, 181, 141, 127 | C_12_H_22_O_4_ | Dodecanedioic acid | Fatty acid | + | + |
| 83 | 4.08 | 309.2057 | -4.5 | 291, 269, 241, 185 | C_18_H_30_O_4_ | Hydroxy oxo-octadecadienoic acid | Fatty acid | + | + |
| 63 | 4.14 | 577.2661 | -2.9 | 225, 81 | C_27_H_46_O_11_S | SQMG(18:3) | Lipid | - | + |
| 84 | 4.16 | 171.0068 | -6.7 | 143, 123, 93 | C_3_H_9_O_6_P | Glycerol-phosphate | Lipid | + | + |
| 85 | 4.24 | 331.2474 | -4.7 | 291, 251, 185, 143, 131 | C_18_H_36_O_5_ | Trihydroxy octadecanoic acid | Fatty acid | + | + |
| 86 | 4.42 | 311.2212 | -5.0 | 223, 208, 151, 128, 87, 57 | C_18_H_32_O_4_ | Dihydroxy octadecadienoic acid | Fatty acid | + | - |
| 87 | 4.61 | 327.2166 | -4.5 | 318, 278, 236, 194, 171, 97 | C_18_H_32_O_5_ | Dihydroxy oxo-octadecenoic acid | Fatty acid | - | + |
| 88 | 4.69 | 593.271 | 4.8 | 277, 241, 152, 78 | C_27_H_47_O_12_P | PI(18:3/0:0) | Lipid | + | - |
| 89 | 4.77 | 329.232 | -3.7 | 293, 201, 171 | C_18_H_34_O_5_ | Hydroxy octadecanedioic acid | Fatty acid | + | + |
| 90 | 4.94 | 275.2009 | -2.6 | 231, 214, 187, 168 | C_18_H_28_O_2_ | Stearidonic acid | Fatty acid | + | + |
| 91 | 4.96 | 293.2111 | -6.7 | 273, 233, 207, 193, 179, 149, 119 | C_18_H_30_O_3_ | Hydroxy octadecatrienoic acid | Fatty acid | + | + |
| 92 | 4.97 | 571.2866 | 3.9 | 255, 241, 223, 152, 96, 78 | C_25_H_49_O_12_P | PI(16:0/0:0) | Lipid | + | + |
| 93 | 5.01 | 277.2161 | -4.2 | 269, 248, 226, 160, 124, 108 | C_18_H_29_O_2_ | Linolenic acid | Fatty acid | + | + |
| 94 | 5.1 | 555.2826 | 5.8 | 225, 81 | C_25_H_48_O_11_S | SQMG(16:0) | Lipid | + | + |
| 95 | 5.15 | 295.2265 | -4.5 | 277, 227, 205, 171, 155, 97 | C_18_H_32_O_3_ | Hydroxy linoleic acid | Fatty acid | + | + |
| 96 | 5.22 | 279.1220 | -7.4 | 235, 219, 177, 149, 137, 113, 103 | C_15_H_20_O_5_ | Hydroxy abscisic acid | Sesquiterpene | + | - |
| 97 | 5.28 | 483.2704 | -5.0 | 255, 152, 78 | C_22_H_45_O_9_P | PG(16:0/0:0) | Lipid | + | - |
| 98 | 5.7 | 271.2266 | -1.5 | 225, 170, 140 | C_18_H_32_O_3_ | Hydroxy palmitic acid | Fatty acid | + | + |
| 99 | 5.73 | 313.2014 | -2.0 | 257, 208, 171, 142, 99 | C_17_H_30_O_5_ | Trihydroxy heptadecadienoic acid | Fatty acid | + | - |
| 100 | 5.82 | 255.2312 | -3.9 | 237, 214, 195, 167, 92 | C_16_H_32_O_2_ | Palmitic acid | Fatty acid | + | + |
| 101 | 6.1 | 279.2319 | -2.5 | 201, 175, 136, 117 | C_18_H_32_O_2_ | Linoleic acid | Fatty acid | - | + |
| 102 | 6.13 | 283.2634 | -2.9 | 279, 205, 163, 138 | C_18_H_36_O_2_ | Stearic acid | Fatty acid | + | + |
| 103 | 6.33 | 671.4629 | -6.1 | 279, 255, 152, 78 | C_37_H_69_O_8_ P | PA(18:2/16:0) | Lipid | + | - |
| 104 | 6.33 | 835.5306 | 3.8 | 579, 553, 417, 409, 391, 315, 297, 281, 259, 255, 241, 223, 152, 96, 78 | C_43_H_81_O_13_P | PI(16:0/18:1) | Lipid | + | + |
| 105 | 6.61 | 861.5471 | -3.2 | 297, 283, 279, 259, 241, 223, 152, 78 | C_45_H_83_O_13_P | PI(18:0/18:2) | Lipid | + | - |
| 106 | 6.79 | 691.4901 | 9.7 | 277, 152, 78 | C_39_H_65_O_8_P | PA(18:3/18:3) | Lipid | - | + |
| 107 | 6.93 | 381.3361 | -3.4 | 335, 279, 224 | C_24_H_46_O_3_ | Hydroxy tetracosenoic acid | Fatty acid | - | + |
| 108 | 7.09 | 833.5155 | -3.7 | 315, 279, 255, 241, 152, 78 | C_43_H_79_O_13_P | PI(16:0/18:2) | Lipid | + | + |
| 109 | 7.23 | 383.3511 | 5.1 | 337, 245, 137, 91 | C_24_H_48_O_3_ | Hydroxy tetracosanoic acid | Fatty acid | + | - |
| 110 | 7.32 | 831.5154 | 6.7 | 277, 255, 241, 152, 96, 78 | C_43_H_77_O_13_P | PI(16:0/18:3) | Lipid | + | - |

Note. PA, phosphatidic acid; PG, phosphatidyl glycerol; PI, phosphatidyl inositol; and SQMG, sulfoquinovosyl monoacylglycerol.

**Table S3.** Differentiating metabolites between the Romanesco (RB) and purple broccoli (PB) samples as identified from the volcano plot of their LC/MS data

| **Metabolite** | **Fold change (FC)** | **Log_2_ (FC)** | **p-value** | **-Log_10_ (p-value)** |
| --- | --- | --- | --- | --- |
| **Metabolites up-regulated in purple broccoli (PB)** | | | | |
| Kaempferol 7-glucoside | 9.8512 | 3.3003 | 9.95E-14 | 13.002 |
| Caffeic acid | 9.4132 | 3.2347 | 2.41E-14 | 13.617 |
| N-Glycosyl asparagine | 9.2698 | 3.2125 | 1.37E-09 | 8.8621 |
| Glucobrassicin | 7.3686 | 2.8814 | 1.34E-10 | 9.8743 |
| Hydroxy benzoic acid glucoside | 5.061 | 2.3394 | 2.87E-09 | 8.542 |
| 3-p-Coumaroyl quinic acid | 5.0446 | 2.3347 | 9.91E-10 | 9.0039 |
| Methyl citric acid | 5.0439 | 2.3345 | 9.41E-10 | 9.0264 |
| Glycerophospho-ethanolamine | 5.0439 | 2.3345 | 9.41E-10 | 9.0264 |
| PA(18:2/16:0) | 5.0439 | 2.3345 | 9.41E-10 | 9.0264 |
| Isorhamnetin 3-O-feruloyl sophoroside | 5.0439 | 2.3345 | 9.41E-10 | 9.0264 |
| Isorhamnetin 3-sinapoyl sophoroside | 5.0439 | 2.3345 | 9.41E-10 | 9.0264 |
| Ferulic acid glucoside | 5.0357 | 2.3322 | 4.68E-10 | 9.3293 |
| PA(18:3/18:3) | 5.0357 | 2.3322 | 4.68E-10 | 9.3293 |
| Quercetin 3-rutinoside-7-glucoside | 5.0357 | 2.3322 | 4.68E-10 | 9.3293 |
| Kaempferol 3-feruloyl sophoroside | 5.0357 | 2.3322 | 4.68E-10 | 9.3293 |
| Quercetin 3-sophoroside 7-glucoside | 5.0357 | 2.3322 | 4.68E-10 | 9.3293 |
| Quercetin 3-feruloyl sophoroside 7-glucoside | 5.0357 | 2.3322 | 4.68E-10 | 9.3293 |
| Glycerol-phosphate | 5.0089 | 2.3245 | 3.26E-12 | 11.487 |
| Hydroxy tetracosenoic acid | 5.0068 | 2.3239 | 1.15E-12 | 11.938 |
| Citric acid | 5.0051 | 2.3234 | 4.29E-13 | 12.368 |
| Quercetin 3-caffeoyl sophoroside 7-glucoside | 5.0052 | 2.3234 | 4.48E-13 | 12.349 |
| Hydroxy tetracosanoic acid | 5.0037 | 2.323 | 1.44E-13 | 12.84 |
| PI(16:0/18:1) | 5.0038 | 2.323 | 1.51E-13 | 12.822 |
| Kaempferol 3-coumaroyl sophoroside 7-glucoside | 5.0032 | 2.3229 | 9.66E-14 | 13.015 |
| SQMG(18:3) | 5.0035 | 2.3229 | 1.23E-13 | 12.909 |
| Kaempferol 3-sophoroside | 5.003 | 2.3228 | 8.07E-14 | 13.093 |
| Isorhamnetin 3-sophoroside | 5.0025 | 2.3227 | 4.13E-14 | 13.384 |
| Feruloyl malate | 5.002 | 2.3225 | 1.69E-14 | 13.773 |
| Caffeic acid glucoside | 5.0018 | 2.3224 | 1.32E-14 | 13.878 |
| Linoleic acid | 5.0012 | 2.3223 | 2.97E-15 | 14.528 |
| Kaempferol 3-sinapoyl sophoroside 7-glucoside | 5.0012 | 2.3223 | 2.97E-15 | 14.528 |
| Quercetin 3-sophoroside | 5.0006 | 2.3221 | 2.10E-16 | 15.677 |
| Leucine | 5.0006 | 2.3221 | 2.40E-16 | 15.62 |
| Kaempferol 3-sophorotrioside | 5.0002 | 2.322 | 4.83E-18 | 17.316 |
| Rutin | 5.0003 | 2.322 | 5.25E-17 | 16.28 |
| Histidine | 5.0004 | 2.322 | 6.51E-17 | 16.186 |
| Isorhamnetin 3-sophoroside 7-glucoside | 5.0004 | 2.322 | 6.51E-17 | 16.186 |
| Glucoiberin | 5.0001 | 2.3219 | 3.89E-19 | 18.411 |
| Neoglucobrassicin | 4.3688 | 2.1272 | 2.28E-15 | 14.642 |
| 4-Methylpentyl glucosinolate | 4.2302 | 2.0807 | 5.68E-09 | 8.2458 |
| Trihydroxy octadecanoic acid | 3.6569 | 1.8706 | 8.63E-09 | 8.064 |
| Chlorogenic acid | 2.3097 | 1.2077 | 7.95E-08 | 7.0997 |
| Kaempferol 3-caffeoyl sophoroside 7-glucoside | 2.214 | 1.1466 | 9.57E-08 | 7.0191 |
| **Metabolites up-regulated in Romanesco broccoli (RB)** | | | | |
| Dihydroxy octadecadienoic acid | 0.1974 | -2.3408 | 3.76E-09 | 8.4244 |
| Stearic acid | 0.19888 | -2.33 | 2.02E-10 | 9.6948 |
| PG(16:0/0:0) | 0.19888 | -2.33 | 2.02E-10 | 9.6948 |
| PI(18:3/0:0) | 0.19888 | -2.33 | 2.02E-10 | 9.6948 |
| PI(16:0/18:3) | 0.19941 | -2.3262 | 2.18E-11 | 10.662 |
| PI(18:0/18:2) | 0.19982 | -2.3233 | 2.96E-13 | 12.528 |
| Abscisic acid | 0.1998 | -2.3233 | 3.74E-13 | 12.427 |
| p-Coumaroyl malate | 0.19982 | -2.3232 | 2.66E-13 | 12.575 |
| Glutamine | 0.19993 | -2.3224 | 1.04E-14 | 13.982 |
| Sinapic acid | 0.19999 | -2.322 | 2.65E-17 | 16.576 |
| Ferulic acid | 0.19998 | -2.322 | 7.86E-17 | 16.105 |
| PI(16:0/18:2) | 0.27904 | -1.8414 | 1.96E-11 | 10.707 |
| Stearidonic acid | 0.29139 | -1.779 | 2.66E-09 | 8.5757 |
| N-Methyl alanine | 0.3427 | -1.545 | 3.96E-09 | 8.4018 |
| Hydroxy oxo-octadecadienoic acid | 0.36018 | -1.4732 | 2.42E-09 | 8.6159 |
| Nonanedioic acid | 0.37161 | -1.4281 | 4.62E-13 | 12.336 |
| Hydroxy abscisic acid | 0.48006 | -1.0587 | 1.91E-08 | 7.7193 |

**Table S4.** 3D interactions and pocket positioning for 3-*p*-coumaroyl quinic acid, glucobrassicin, and rutin towards the AChE and BuChE target enzymes.

| **Comp.** | **Receptor** | **3D interactions** | **3D positioning** |
| --- | --- | --- | --- |
| **3-*p*-Coumaroyl quinic acid** | **AChE** | 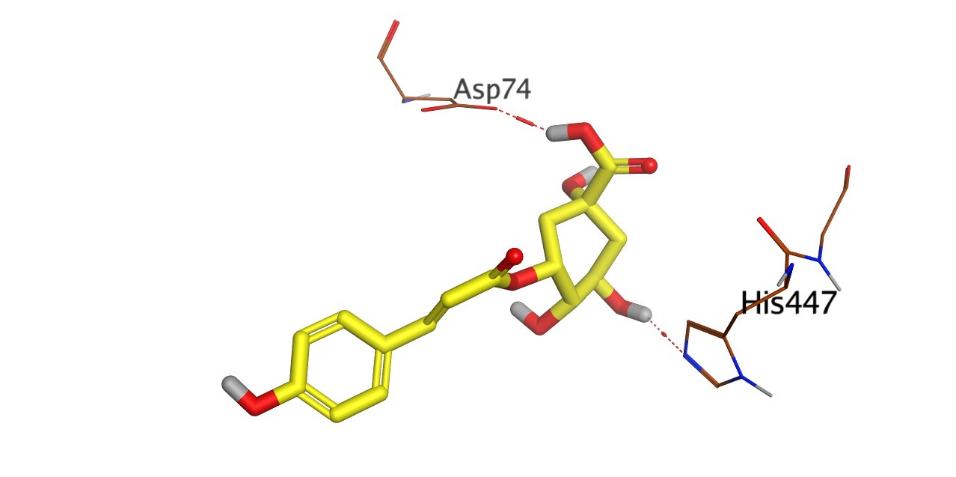 | 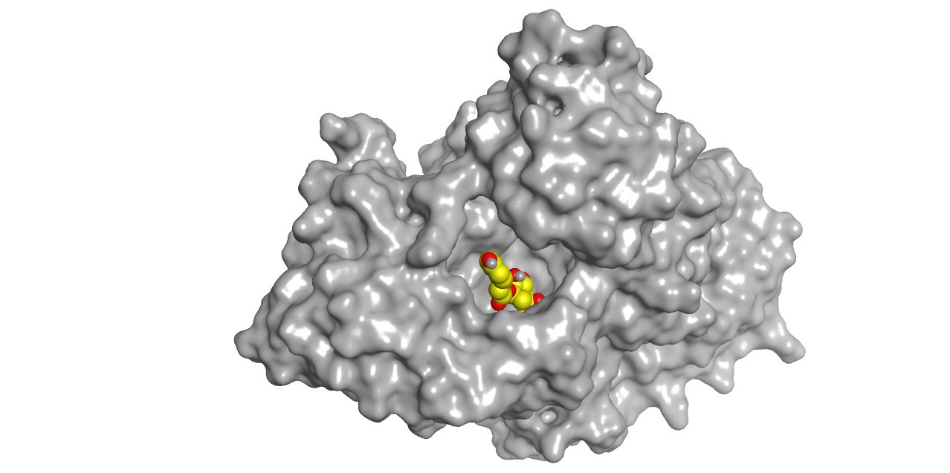 |
|  | **BuChE** | 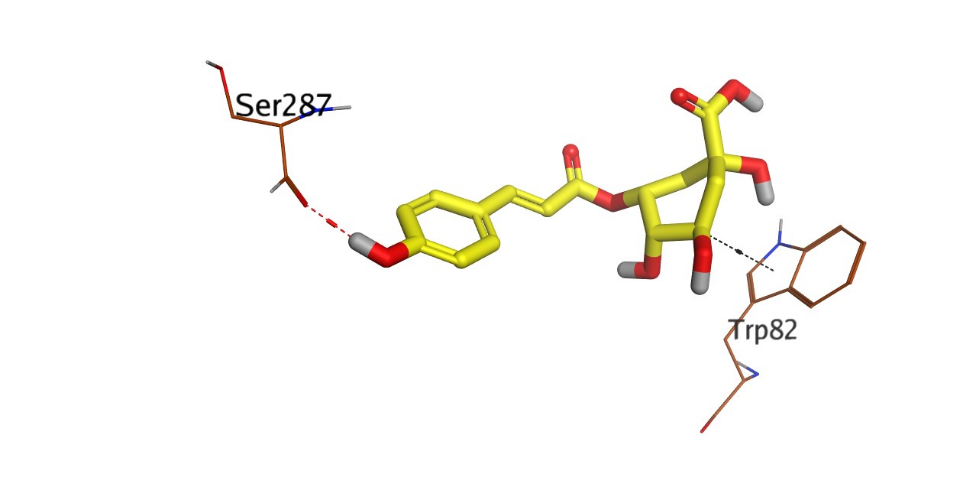 | 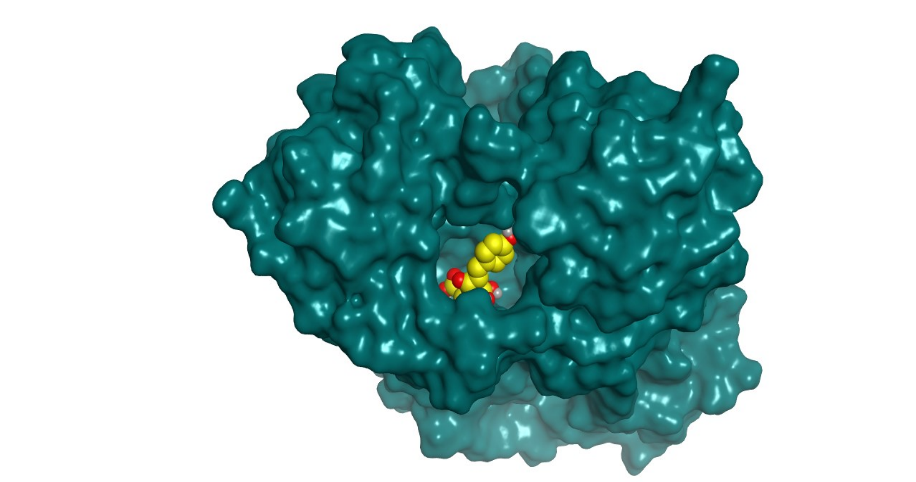 |
| **Glucobrassicin** | **AChE** | 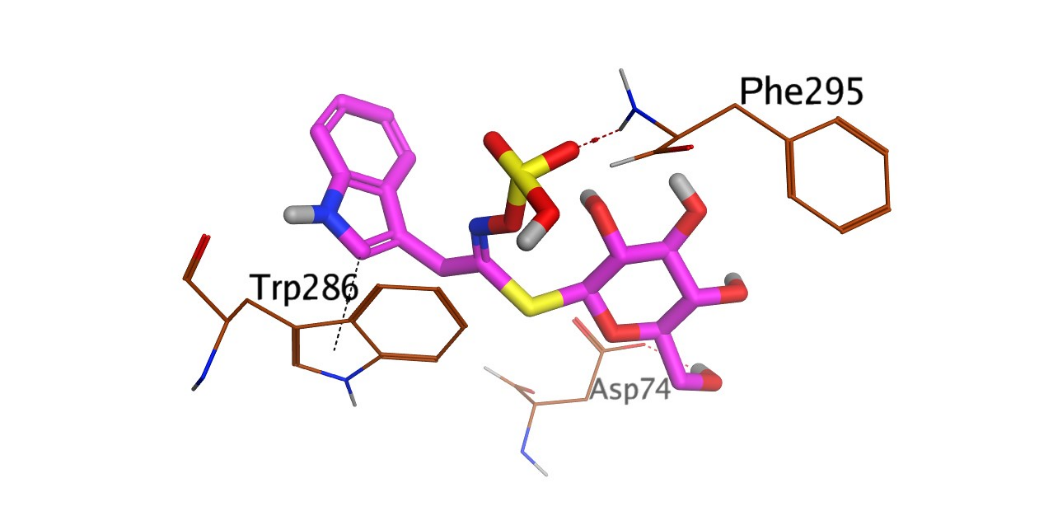 | 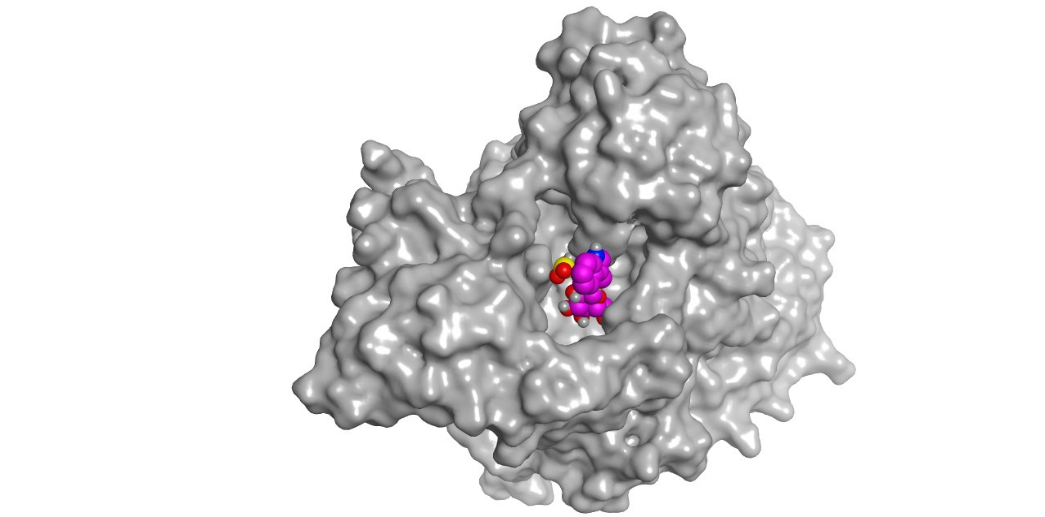 |
|  | **BuChE** | 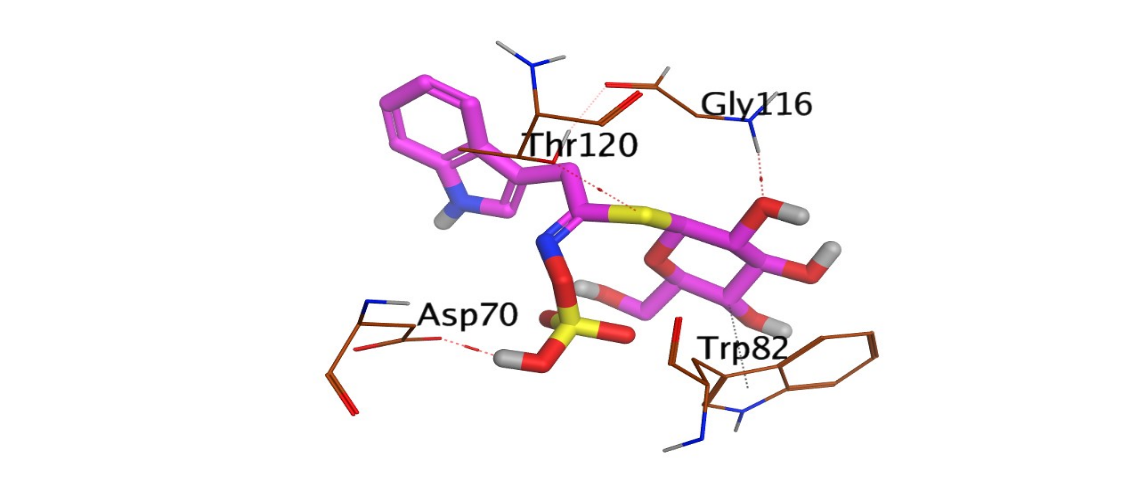 | 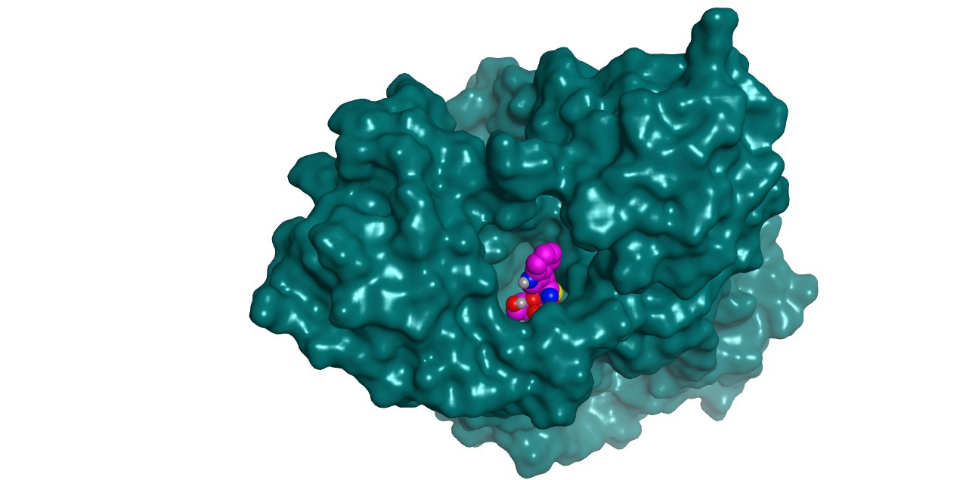 |
| **Rutin** | **AChE** | 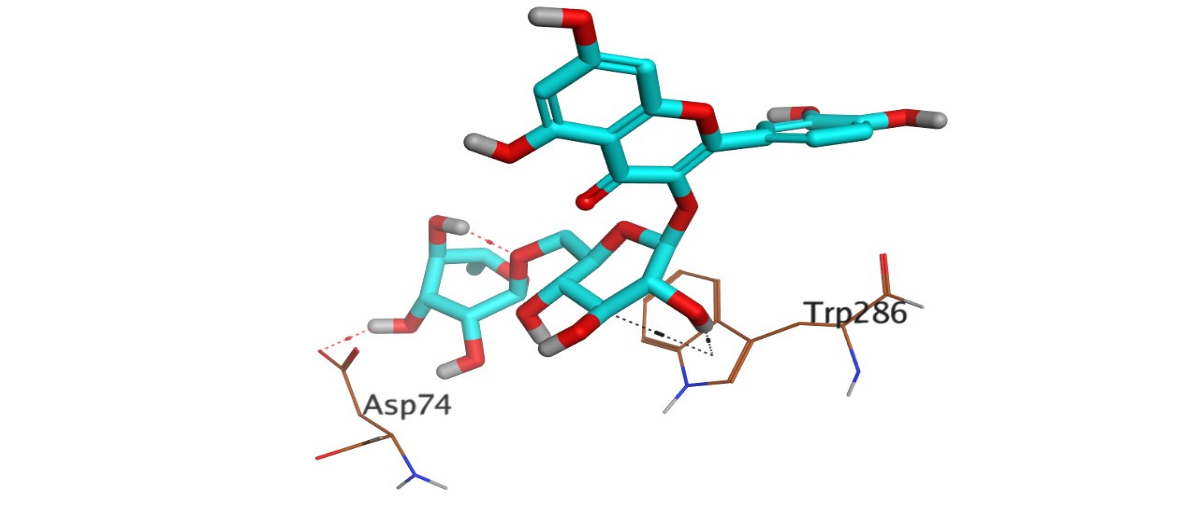 | 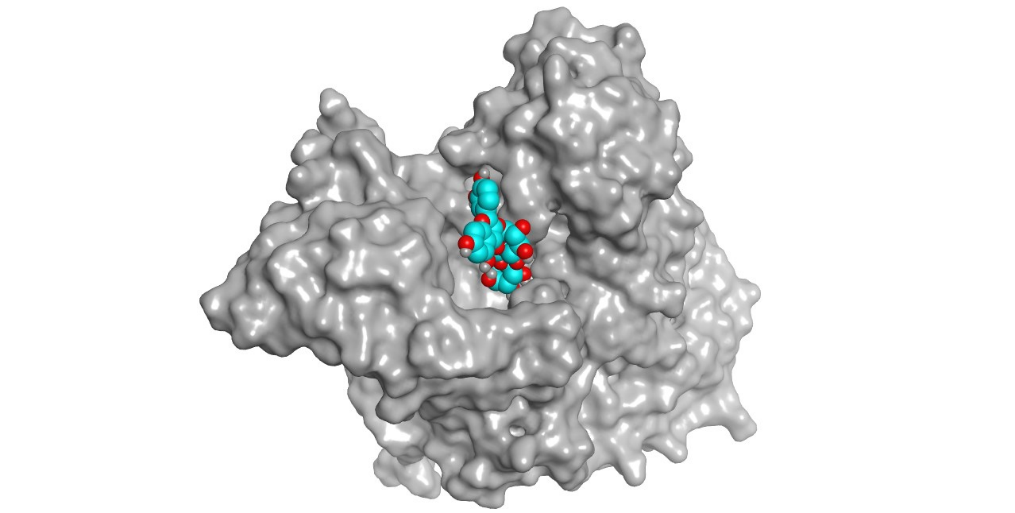 |
|  | **BuChE** | 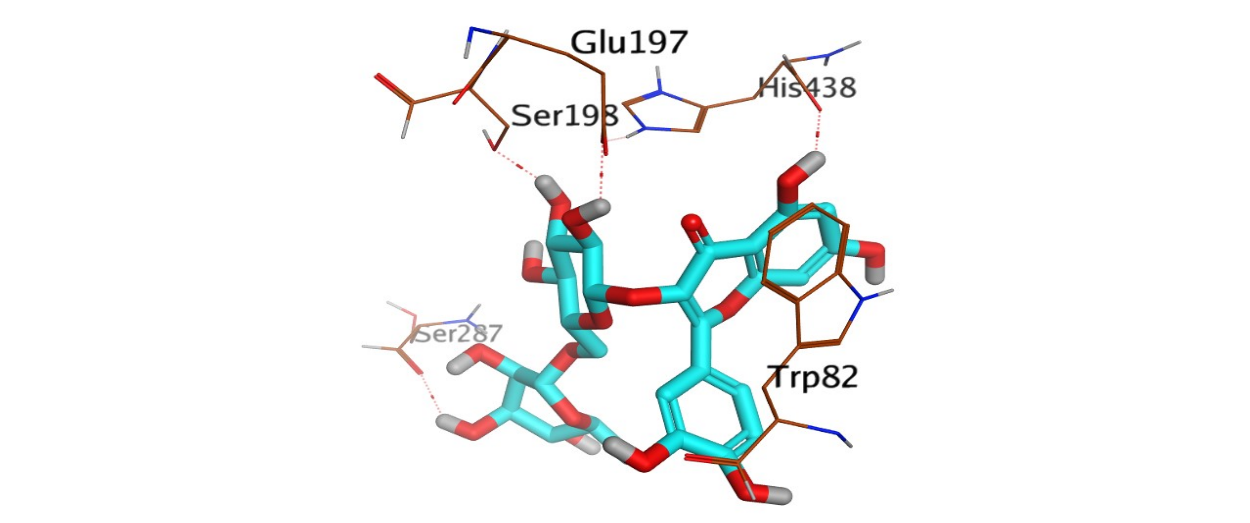 | 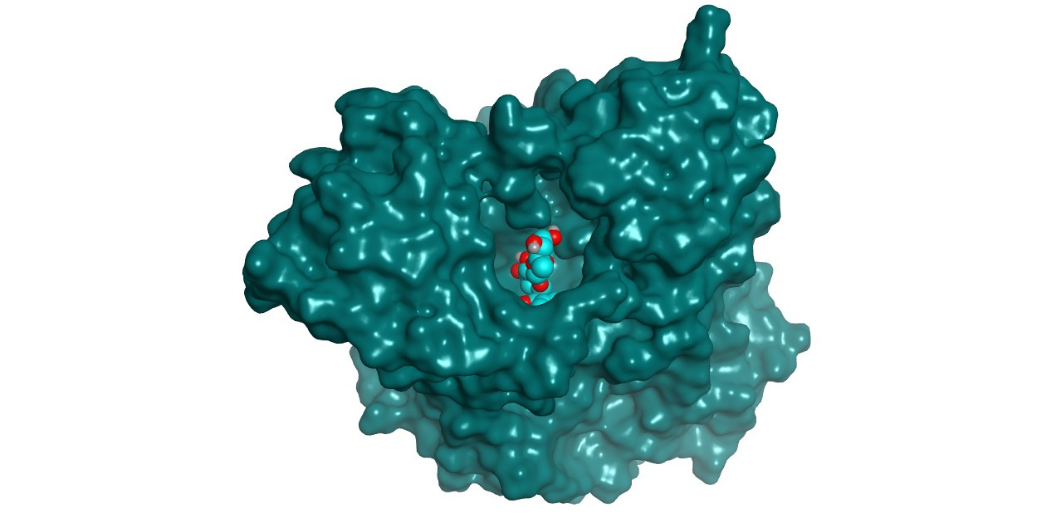 |

**References**

1. Kim H, Moon JY, Kim H, Lee D-S, Cho M, Choi H-K, Kim YS, Mosaddik A, Cho SK (2010) Antioxidant and antiproliferative activities of mango (*Mangifera indica* L.) flesh and peel. Food Chem 121(2):429-436. https://doi.org/https://doi.org/10.1016/j.foodchem.2009.12.060

2. Benzie IF, Strain JJ (1996) The ferric reducing ability of plasma (FRAP) as a measure of “antioxidant power”: the FRAP assay. Anal Biochem 239(1):70-76. https://doi.org/https://doi.org/10.1006/abio.1996.0292

3. Rahman MM, Islam MB, Biswas M, Khurshid Alam A (2015) In vitro antioxidant and free radical scavenging activity of different parts of *Tabebuia pallida* growing in Bangladesh. BMC Res Notes 8(1):1-9. https://doi.org/https://doi.org/10.1186/s13104-015-1618-6

4. Srour AM, Dawood DH, Nossier ES, El-Shiekh RA, Mahmoud AE, Hussien AG, Omran MM, Ali MM (2022) Design, Synthesis and Molecular Docking Simulation of Oxindole-Based Derivatives with Dual VEGFR-2 and Cholinesterase Inhibitory Activities. J Mol Struct:134130. https://doi.org/https://doi.org/10.1016/j.molstruc.2022.134130

5. Fawazy NG, Panda SS, Mostafa A, Kariuki BM, Bekheit MS, Moatasim Y, Kutkat O, Fayad W, El-Manawaty MA, Soliman AA (2022) Development of spiro-3-indolin-2-one containing compounds of antiproliferative and anti-SARS-CoV-2 properties. Sci Rep 12(1):1-21. https://doi.org/https://doi.org/10.1038/s41598-022-17883-9

6. Tian Q, Rosselot RA, Schwartz SJ (2005) Quantitative determination of intact glucosinolates in broccoli, broccoli sprouts, Brussels sprouts, and cauliflower by high-performance liquid chromatography–electrospray ionization–tandem mass spectrometry. Anal Biochem 343(1):93-99. https://doi.org/https://doi.org/10.1016/j.ab.2005.04.045

7. Ares AM, Nozal MJ, Bernal JL, Bernal J (2014) Optimized extraction, separation and quantification of twelve intact glucosinolates in broccoli leaves. Food Chem 152:66-74. https://doi.org/https://doi.org/10.1016/j.foodchem.2013.11.125

8. Salas-Millán J-Á, Aznar A, Conesa E, Conesa-Bueno A, Aguayo E (2022) Functional food obtained from fermentation of broccoli by-products (stalk): Metagenomics profile and glucosinolate and phenolic compounds characterization by LC-ESI-QqQ-MS/MS. Lwt 169:113915. https://doi.org/https://doi.org/10.1016/j.lwt.2022.113915

9. Lin L-Z, Harnly JM (2009) Identification of the phenolic components of collard greens, kale, and Chinese broccoli. J Agric Food Chem 57(16):7401-7408. https://doi.org/https://doi.org/10.1021/jf901121v

10. Vallejo F, Tomás-Barberán F, Ferreres F (2004) Characterisation of flavonols in broccoli (*Brassica oleracea* L. var. *italica*) by liquid chromatography–UV diode-array detection–electrospray ionisation mass spectrometry. J Chromatogr A 1054(1-2):181-193. https://doi.org/https://doi.org/10.1016/j.chroma.2004.05.045

11. Gratacós-Cubarsí M, Ribas-Agusti A, García-Regueiro JA, Castellari M (2010) Simultaneous evaluation of intact glucosinolates and phenolic compounds by UPLC-DAD-MS/MS in *Brassica oleracea* L. var. *botrytis*. Food Chem 121(1):257-263. https://doi.org/https://doi.org/10.1016/j.foodchem.2009.11.081

12. Ibrahim RM, Elmasry GF, Refaey RH, El-Shiekh RA (2022) *Lepidium meyenii* (maca) roots: UPLC-HRMS, molecular docking, and molecular dynamics. ACS omega 7(20):17339-17357. https://doi.org/https://doi.org/10.1021/acsomega.2c01342

13. Khattab M, Al-Karmalawy AA (2021) Computational repurposing of benzimidazole anthelmintic drugs as potential colchicine binding site inhibitors. Future Med Chem 13(19):1623-1638. https://doi.org/10.4155/fmc-2020-0273

14. Taher RF, Al-Karmalawy AA, Abd El Maksoud AI, Khalil H, Hassan A, El-Khrisy E-DA, El-Kashak W (2021) Two new flavonoids and anticancer activity of Hymenosporum flavum: in vitro and molecular docking studies. J Herbmed Pharmacol 10(4):443-458. https://doi.org/10.34172/jhp.2021.52

15. Elmaaty AA, Darwish KM, Chrouda A, Boseila AA, Tantawy MA, Elhady SS, Shaik AB, Mustafa M, Al-karmalawy AA (2022) In Silico and In Vitro Studies for Benzimidazole Anthelmintics Repurposing as VEGFR-2 Antagonists: Novel Mebendazole-Loaded Mixed Micelles with Enhanced Dissolution and Anticancer Activity. ACS Omega 7(1):875-899. https://doi.org/10.1021/acsomega.1c05519

16. Elagawany M, Elmaaty AA, Mostafa A, Abo Shama NM, Santali EY, Elgendy B, Al-Karmalawy AA (2022) Ligand-based design, synthesis, computational insights, and in vitro studies of novel N-(5-Nitrothiazol-2-yl)-carboxamido derivatives as potent inhibitors of SARS-CoV-2 main protease. J Enzyme Inhib Med Chem 37(1):2112-2132. https://doi.org/10.1080/14756366.2022.2105322

17. Al-Karmalawy AA, El-Gamil DS, El-Shesheny R, Sharaky M, Alnajjar R, Kutkat O, Moatasim Y, Elagawany M, Al-Rashood ST, Binjubair FA, Eldehna WM, Noreddin AM, Zakaria MY (2023) Design and statistical optimisation of emulsomal nanoparticles for improved anti-SARS-CoV-2 activity of N-(5-nitrothiazol-2-yl)-carboxamido candidates: in vitro and in silico studies. J Enzyme Inhib Med Chem 38(1):2202357. https://doi.org/10.1080/14756366.2023.2202357

18. Elmaaty AA, Eldehna WM, Khattab M, Kutkat O, Alnajjar R, El-Taweel AN, Al-Rashood ST, Abourehab MAS, Binjubair FA, Saleh MA, Belal A, Al-Karmalawy AA (2022) Anticoagulants as potential SARS-CoV-2 Mpro inhibitors for COVID-19 patients: In vitro, molecular docking, molecular dynamics, DFT, and SAR studies. Int J Mol Sci 23(20):12235. https://doi.org/https://doi.org/10.3390/ijms232012235
